# *Cinnamomum cassia* (L.) J.Presl Alleviates Allergic Responses in Asthmatic Mice *via* Suppression of MAPKs and MMP-9

**DOI:** 10.3389/fphar.2022.906916

**Published:** 2022-08-11

**Authors:** Je-Oh Lim, Yun Hee Kim, Ik Soo Lee, Woong-Il Kim, Se-Jin Lee, So-Won Pak, In-Sik Shin, Taesoo Kim

**Affiliations:** ^1^ College of Veterinary Medicine and BK21 FOUR Program, Chonnam National University, Gwangju, South Korea; ^2^ KM Convergence Research Division, Korea Institute of Oriental Medicine, Daejeon, South Korea; ^3^ R&D Strategy Division, Korea Institute of Oriental Medicine, Daejeon, South Korea

**Keywords:** *Cinnamomum cassia* (L.) J.Presl, asthma, MAPKs, MMP-9, mice (balb/c)

## Abstract

The prevalence of asthma is gradually increasing, and endangers human health. Many therapeutic agents have been developed to address this concern. *Cinnamomum cassia* (L.) J.Presl is a traditional herbal remedy in China, Japan, and Korea and used mainly to control common cold, cough, pneumonitis and fever in Donguibogam, a medical encyclopedia of Korea. Therefore, we investigated whether *C. cassia* (L.) J.Presl extract (CCE) confers protective effects on asthma model induced by ovalbumin (OVA). The animals were received intraperitoneal administration of OVA on day 1 and 14, and then subjected to OVA inhalation from day 21–23. They were orally treated CCE (30 and 100 mg/kg) from day 18–23. CCE administration decreased allergic responses, including airway hyperresponsiveness, eosinophilia, inflammatory cytokine production, and immunoglobulin E in OVA-exposed mice, along with the decline in inflammatory cell count and mucus secretion in respiratory tract. Additionally, CCE suppressed MAPK phosphorylation and MMP-9 expression in OVA-exposed mice. Overall, CCE treatment attenuated allergic responses induced by OVA exposure, which may be connected to the suppression of MAPK phosphorylation.

## Introduction

Asthma is a chronic inflammatory disorder of respiratory tract that causes recurrent clinical symptoms such as difficulty in breathing, coughing, and heavy breathing (Rehman et al., 2018). About 235 million people suffered from asthma worldwide (Kumar and Ram, 2017) and its prevalence continues to rise with the increase in allergens in the air, including air-pollutants, chemicals, and pollen (Chatkin et al., 2021). Onset of asthma is induced by inhalation of allergens, which induces airway hyperresponsiveness (AHR), shortness of breath, sputum and cough *via* allergic responses induced by the elevation of inflammatory mediators (Bates, 2016; Bhalla et al., 2018; Cloutier et al., 2020). Currently, various treatment options are being researched, and many researchers have been studied to find therapeutic materials for treatment of asthma (Cloutier et al., 2020). Although leukotriene receptor antagonists, beta-adrenergic receptor agonists, corticosteroids and immunomodulatory drugs have been used in clinical settings, they have limited potential for asthma treatment since they lead to adverse effects or may only improve clinical symptoms (Aranez and Ambrus, 2020). Therefore, the social demand for the development of asthma treatment is on the rise. Mitogen-activated protein kinase (MAPK) pathway is a key process in the onset and progression of inflammatory responses in respiratory tract. MAPKs (such as extracellular signal regulated kinase 1/2 (ERK1/2), c-Jun N-terminal kinase (JNK), and p-38) are phosphorylated by various stimuli and induce the activation of immune cells and production of inflammatory mediators including cytokines and chemokines by activation of various transcriptional factors related to inflammatory responses (Santana et al., 2019). At the onset and development of asthma, the phosphorylation of MAPKs also leads to inflammatory cell infiltration into respiratory tract *via* increased production of cytokines and immunoglobulin E (IgE) (Johnson and Lapadat, 2002; Chialda et al., 2005; Junttila et al., 2008). In addition, MAPKs are closely involved in the activation and expression of matrix-metalloproteinases (MMPs) connected with airway remodeling and airway inflammation in the development of asthma (Shin et al., 2017). Considering these factors, the modulation of MAPK signaling is an important target to control asthma.

*Cinnamomum cassia* (L.) J.Presl is a traditional oriental medicine commonly applied to treating arthritis, common cold, cough, gastrointestinal disorders, and osteoporosis (Zhang et al., 2019). In Donguibogam, a medical encyclopedia written by Heo Jun, a royal physician of Korea in the 17th century, *C. cassia* (L.) J.Presl was mainly used to control common cold, cough, pneumonitis and fever. Additionally, in modern medicine, it also exhibits therapeutic effects on cancer, diabetes, and viral infection (Fatima et al., 2016; Lin et al., 2017; Kaur et al., 2018). A lot of research has focused on its powerful anti-inflammatory functions (Yang et al., 2017; Liu et al., 2020; Lee and Lim, 2021), which make *C. cassia* (L.) J.Presl a viable therapeutic candidate for effective reduction of airway inflammation in asthma. However, this candidacy for asthma treatment/management has not been tested in laboratory or clinical settings, yet.

We performed an experimental study to assess the potential of *C. cassia* (L.) J.Presl in treating asthma using an asthma model caused by ovalbumin (OVA) in mice. In order to explore its mechanism of action, the protein expression altered by *C. cassia* (L.) J.Presl was investigated, with a focus on MAPK signaling.

## Materials and Methods

### Plant Material

*C. cassia* (L.) J.Presl was purchased from an herbal medicine store (Omniherb, Yeongcheon, South Korea), and identified and authenticated by an herbarium botanist. A voucher specimen was deposited in the Herbarium of Korea Institute of Oriental Medicine (Daejeon, South Korea).

### Chemical and Reagents

Cinnamaldehyde, cinnamic acid, cinnamyl alcohol, coumarin, and 2-methoxycinnamaldehyde were purchased from Sigma-Aldrich (purity: > 99%, St. Louis, MO). Acetonitrile, methanol, and water purcahsed from J.T. Baker (high-performance liquid chromatography (HPLC) grade, Phillipsburg, NJ). Formic acid was obtained from Merck (Darmstadt, Germany).

### Preparation of Sample and Standard Solution

*C. cassia* (L.) J.Presl (1 kg) was extracted with 70% ethanol (10 L) at 80 °C for 3 h. The extract was filtered, evaporated, and then freeze dried (42.9 g). The yield of extract was 4.3%. *C. cassia* (L.) J.Presl extract (CCE, 100 mg) was dissolved in methanol (10 ml) and the solution was filtered through a syringe filter (0.45 μm, Cytiva, Marlborough, MA) prior to injection. A methanol standard stock solution was contained with 5 reference standards (all at 1 mg/ml) and applied for HPLC analyses after serial dilution.

### HPLC Condition

HPLC analyses were performed using an Agilent 1200 HPLC instrument (Agilent Technologies, Santa Clara, CA). The instrument consisted of an auto-sampler, binary pump, column compartment, diode array detector, and vacuum degasser. The Agilent ChemStation software was used as the data processor. The analytical column was used a Zorbax Eclipse Plus column (250 × 4.6 mm, 5.0 μm; Agilent) and its temperature was kept at 40°C. The mobile phase consisted of 0.1% formic acid in water (A) and acetonitrile (B) with gradient elution. The gradient solvent system was optimized as follows: 95–50% A (0–45 min), 50–0% A (45–50 min), 100% B (50–60 min), and 95% A (61–70 min) at a flow rate of 1.0 ml/min. The detection was performed at 260 nm and the injection volume was 5 μL. Standard solutions at 5 levels were arranged by serially diluting the stock solution to evaluate for linearity. Each analysis was repeated 3 times, and the calibration curves were fitted by linear regression. The limit of detection (LOD) and limit of quantification (LOQ) data collected under the optimal chromatographic conditions were accessed using signal-to-noise (S/N) ratios of 3 and 10, respectively.

### Procedure for OVA-EXPOSED Asthma Model

The BALB/c female mice (6-week old, SAMTAKO Co., Ltd. Osan, South Korea) were kept at relative humidity (55 ± 5%), room temperature (22 ± 2°C), and a 12 h night/day cycle. The animals were allowed sterilized tap wat and standard rodent chow *ad libitum*. This study was approved by CNU Institutional Animal Care and Use Committee (CNU IACUC-YB-2020-99).

To determine experimental dosage of CCE, we performed preliminary study using OVA exposed asthma model. In preliminary study, we evaluated the therapeutic effects of CCE at 100 mg/kg and 200 mg/kg to determine the doses used in this experiment. In the results of preliminary study, CCE treatment (100 mg/kg and 200 mg/kg) reduced the inflammatory cell count in BALF and IgE in serum from OVA exposed mice. However, these reductions were more detected in 100 mg/kg group than 200 mg/kg group. Based on the result of preliminary study, we determined 100 mg/kg, as high dose of CCE. All animals were designed into five groups (*n* = 7) as follows: NC (Normal control, nonasthma + PBS administration), OVA (asthma + PBS administration), DEX (asthma + dexamethasone), CCE 30 and CCE 100 (asthma + CCE 30 mg/kg and 100 mg/kg, respectively). To establish an asthma model, mice were sensitized on day 1 and 14 *via* intraperitoneal administration of OVA (20 μg, Sigma-Aldrich) mixed with aluminum hydroxide (2 mg, Sigma-Aldrich) and then they were exposed to OVA (1%) for 1 h/day from day 21–23. Dexamethasone (2 mg/kg) and CCE (30 mg/kg and 100 mg/kg) were daily treated from day 18–23 by oral gavage. CCE was dissolved in PBS. On day 24, AHR was assessed using noninvasive whole-body plethysmograph (Allmedicus, Seoul, South Korea). It was accessed following methylcholine exposure for 3 min and the results expressed as Penh value. This experimental model is acute asthma model. Although this model has successfully reproduced many characteristics of asthma such as IgE, airway inflammation, goblet cell hyperplasia and AHR, many histopathological characteristics in chronic human asthma including chronic inflammation of the airway wall and remodeling (Aun et al., 2017).

### Evaluation of Bronchoalveolar Lavage Fluid (BALF) and Serun

Whole blood was collected and centrifuged (10 min, 200 × g) to separate the serum on day 25, which was used to measure OVA-specific IgE by ELISA kit (R&D systems Inc., Minneapolis, MN). To obtain BALF, we conducted tracheostomy on the mice as reported previously (Shin et al., 2017). An endotracheal tube was put into the trachea of mouse. Following instillation of PBS (0.7 ml) into the lung tissue, BALF was collected using 2 aspirations (total volume: 1.4 ml). BALF was then centrifuged (10 min, 200 × g) and then its supernatants were collected in new tubes to measure interleukin (IL)-4, IL-5, and IL-13 using ELISA (R&D system Inc.). The remaining pellet was dissolved in PBS (200 µL) and total number of cells of BALF were measured using Cell Countess III (Thermo Fisher Scientific, San Diego, CA). To perform visible inflammatory cell count, the cells were attached to the slides using cytocentrifuge and then stained by Diff-Quik reagent (Thermo Fisher Scientific) and the inflammatory cells were differentially counted using light microscope (Leica, Wetzlar, Germany). The differential inflammatory cell count in BALF was calculated by applying the visible inflammatory cell count to the total cell count from Cell Countess III.

### Western Blotting

To study the protein expression altered by CCE treatment, we conducted Western blotting as a previously described protocol (Shin et al., 2017). The primary antibodies (Cell Signaling Technology, Beverly, MA) were used as follows: p-ERK (1:1000), p-JNK (1:1000), p-p38 (1:1000), t-ERK (1:1000), p38 (1:1000), and t-JNK (1:1000). The intensity of protein band was evaluated using ChemiDoc (Bio-Rad Laboratories, Hercules, CA).

### Histopathology

The left lung tissue was fixed, dehydrated, embedded in paraffin, and sliced into sections (4 µm). These sections were stained with hematoxylin and eosin (H&E, Sigma-Aldrich) to access the degree of inflammatory response and periodic acid-Schiff (PAS, Abcam, Cambridge, United Kingdom) to evaluate mucus secretion of goblet cells. The quantitative analysis of airway inflammation and mucus secretion was performed using an image analyzer (IMT i-solution Inc., Vancouver, BC, Canada). To perform quantitative analysis of inflammation and mucus secretion, we obtained the histological picture (H&E and PAS stained slides) of each animal in experimental groups using digital camera (IMT i-solution Inc.,) In quantitative analysis of inflammation, we evaluated inflammatory response for total area (captured on 200 × magnification). In quantitative analysis of mucus secretion, we evaluated mucus secretion for bronchial area. Quantitative value was expressed as percent (%, inflammation or mucu secretion area vs. arranged area). In addition, to access MMP-9 expression, we conducted immunofluorescence analysis as reported previously (Lim et al., 2021). The slides were mounted using Prolong Gold antifade with 4′,6-diamidino-2-phenylindole (Thermo Fisher Scientific) and evaluated by confocal microscopy (LSM980, Carl Zeiss, Oberkochen, Germany).

### Gelatin Zymography

Gelatin zymography was conducted as reported previously (Shin et al., 2013). The protein of lung tissue was loaded on the gel and electrophoresis was carried out. Further, the gels were washed, incubated (37°C, for 16 h) and then stained with Coomassie brilliant blue (Daejung, Siheung, South Korea). The activity of gelatinase was indicated as white bands on a blue background, representing proteolysis, which was quantified by Chemi-Doc (Bio-Rad Laboratories).

### Statistical Analysis

The results were represented as mean ± standard deviation. Statistical analyses was determined using analysis of variance followed by Dunnett’s test for multiple comparisons. *p* values <0.05 were determined to be significant.

## Results

### HPLC Analysis OF *C. Cassia* (L.) J.PRESL

**FIGURE 1:**
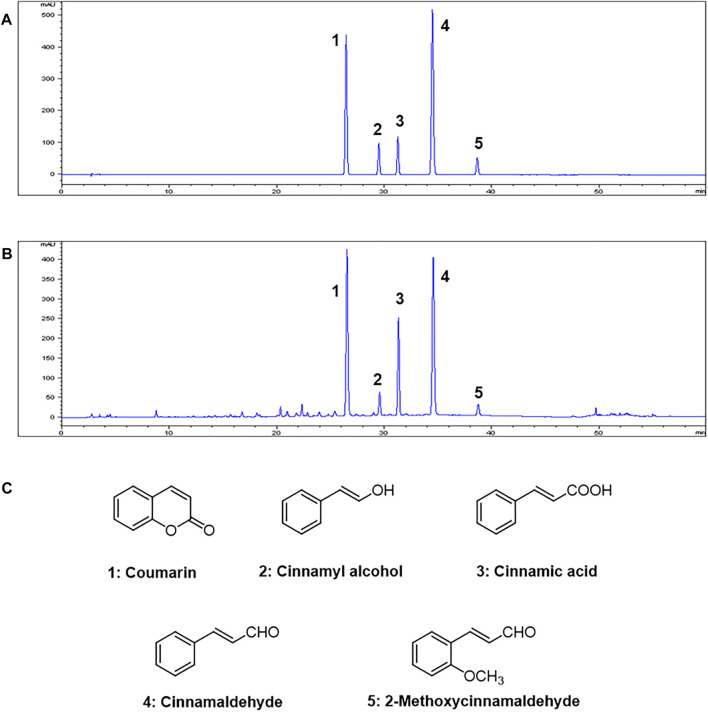
HPLC chromatograms of **(A)** Five standard compound mixtures and **(B)** Ethanolic extract (70%) of *C. cassia* (L.) J.Presl. **(C)** Chemical structures of five compounds. Peak identification: 1, coumarin; 2, cinnamyl alcohol; 3, cinnamic acid; 4, cinnamaldehyde; 5, 2-methoxycinnamaldehyde. Detection was at 260 nm.

HPLC analysis of the 70% ethanol extract of *C. cassia* (L.) J.Presl showed five main peaks (Figures 1A,B). The identifications of peaks 1-5 are coumarin, cinnamyl alcohol, cinnamic acid, cinnamaldehyde, and 2-methoxycinnamaldehyde, respectively (Figure 1C). The regression equations of 5 reference standards were described in Table 1 with the LOD and LOQ values, and the analytical results are shown in Table 2.

**TABLE 1:** Regression equation, linearity, LOD, and LOQ for five marker compounds (*n* = 3).

Compound	Regression equation^a^	Linear Range (μg/ml)	Linearity (*R* ^2^)	LOD^b^ (μg/ml)	LOQ^c^ (μg/ml)
Coumarin	y = 12.448x + 169.54	200–1000	0.9992	2.31	7.01
Cinnamyl alcohol	y = 26.754x + 21.07	20–100	0.9995	0.28	0.85
Cinnamic acid	y = 30.577x + 22.49	20–100	0.9995	0.24	0.74
Cinnamaldehyde	y = 16.135x + 235.68	200–1000	0.9994	2.80	8.49
2-Methoxycinnamaldehyde	y = 8.2612x + 7.57	20–100	0.9996	0.19	0.59

a y, peak area of compound; x, concentration (μg/ml) of compound.

b LOD, limit of detection, S/N = 3.

c LOQ, limit of quantification, S/N = 10.

**TABLE 2:** Contents of five compounds in the 70% ethanol extract of *C. cassia* (L.) J.Presl.

Compound	Content (mean ± SD, *n* = 3)	
	mg/g	%
Coumarin	35.76 ± 0.95	3.58
Cinnamyl alcohol	2.33 ± 0.07	0.23
Cinnamic acid	7.39 ± 0.29	0.74
Cinnamaldehyde	31.89 ± 1.11	3.19
2-Methoxycinnamaldehyde	3.71 ± 0.28	0.37

a Values are presented as means ± SD.

### Effects of CCE on AHR in OVA-EXPOSED Mice

**FIGURE 2:**
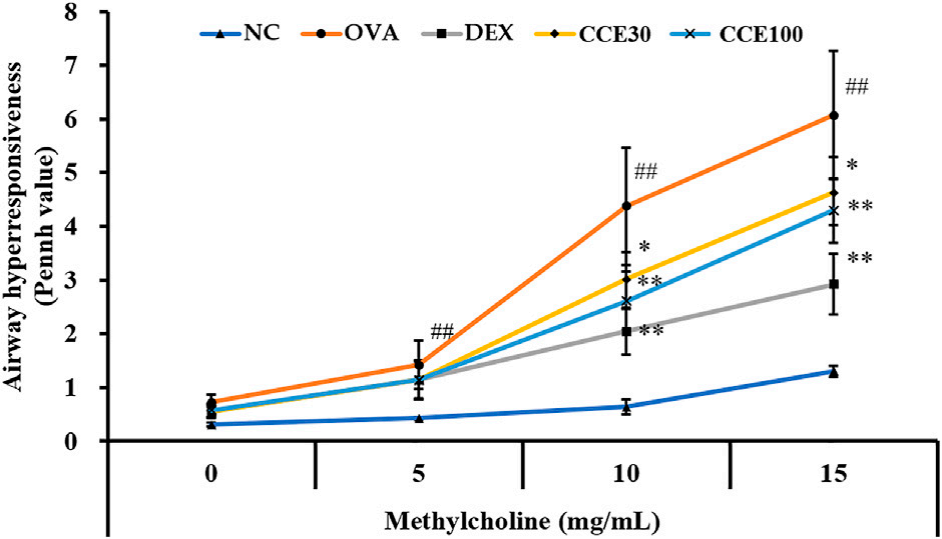
CCE reduced the elevated AHR in OVA-exposed mice. AHR was measured by whole-body plethysmograph. NC, nonasthma and PBS treatment; OVA, asthma and PBS treatment; DEX, asthma and dexamethasone treatment; CCE 30 and CCE 100, asthma and CCE treatment (30 mg/kg and 100 mg/kg, respectively). ##*p* < 0.01, *vs*. NC; ^*^,^**^ *p* < 0.05 and <0.01 *vs*. OVA, respectively (*n* = 7 per group).

The OVA group showed an obvious increase in AHR in comparison with the NC (Figure 2). A marked reduced AHR was seen in the DEX group, compared with the OVA group with the elevation of methacholine’s concentration. Both the CCE groups showed a decline of AHR induced by OVA exposure, which was especially detected in 100 mg/kg group.

### Effects of CCE on Inflammatory Cell Count of BALF in OVA-EXPOSED Mice

**FIGURE 3:**
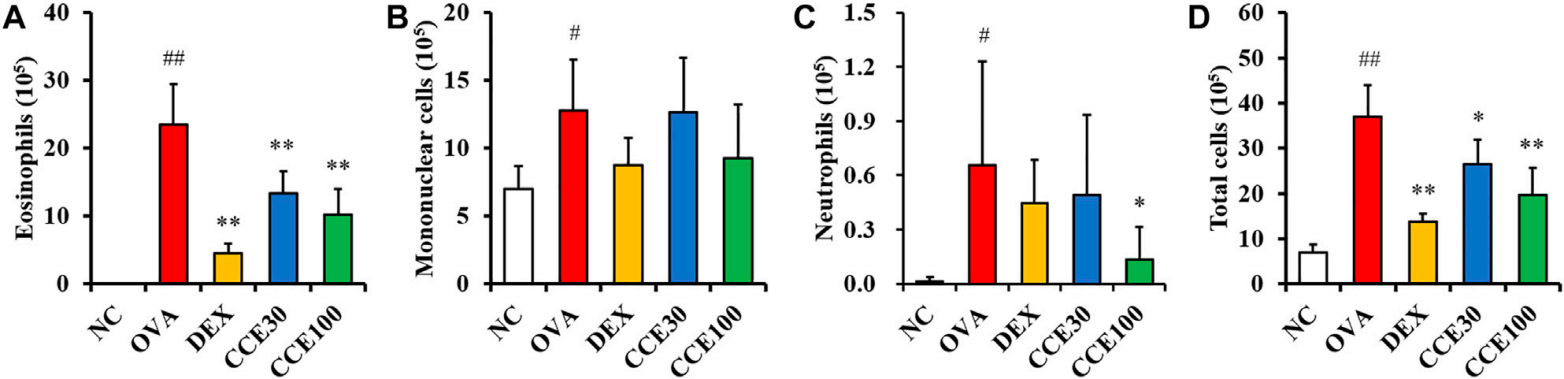
CCE decreased inflammatory cell recruitment in BALF from OVA-exposed mice. **(A)** Eosinophils, **(B)** mononuclear cells, **(C)** neutrophils, **(D)** total cells. NC, nonasthma and PBS treatment; OVA, asthma and PBS treatment; DEX, asthma and dexamethasone treatment; CCE 30 and CCE 100, asthma and CCE treatment (30 mg/kg and 100 mg/kg, respectively). ^#^,^##^ *p* < 0.05 and <0.01, *vs*. NC, respectively; *,**p < 0.05 and <0.01 *vs*. OVA, respectively (*n* = 7 per group).

The eosinophils count in the BALF obviously elevated in the OVA group, compared with the NC (Figure 3A), while a totally contrasting effect was seen in the DEX group, compared with the OVA group. The CCE treated groups displayed a decline in the number of eosinophils in OVA-exposed mice, particularly in the 100 mg/kg group, compared with OVA group. The mononuclear cells and neutrophils count in BALF increased in the OVA group, compared with the NC (Figures 3B,C). Although the CCE treated groups decreased mononuclear cells and neutrophils count, a significant decrease was observed in the high dose in the neutrophils count. Additionally, the OVA group saw a steep rise in the total cell count of BALF in comparison to the NC, whereas in CCE groups, a decline in the total cell count compared with the OVA group was detected (Figure 3D).

### Effects of CCE on Levels of Inflammatory Cytokines and OVA-SPECIFIC IgE in OVA-EXPOSED Mice

**FIGURE 4:**
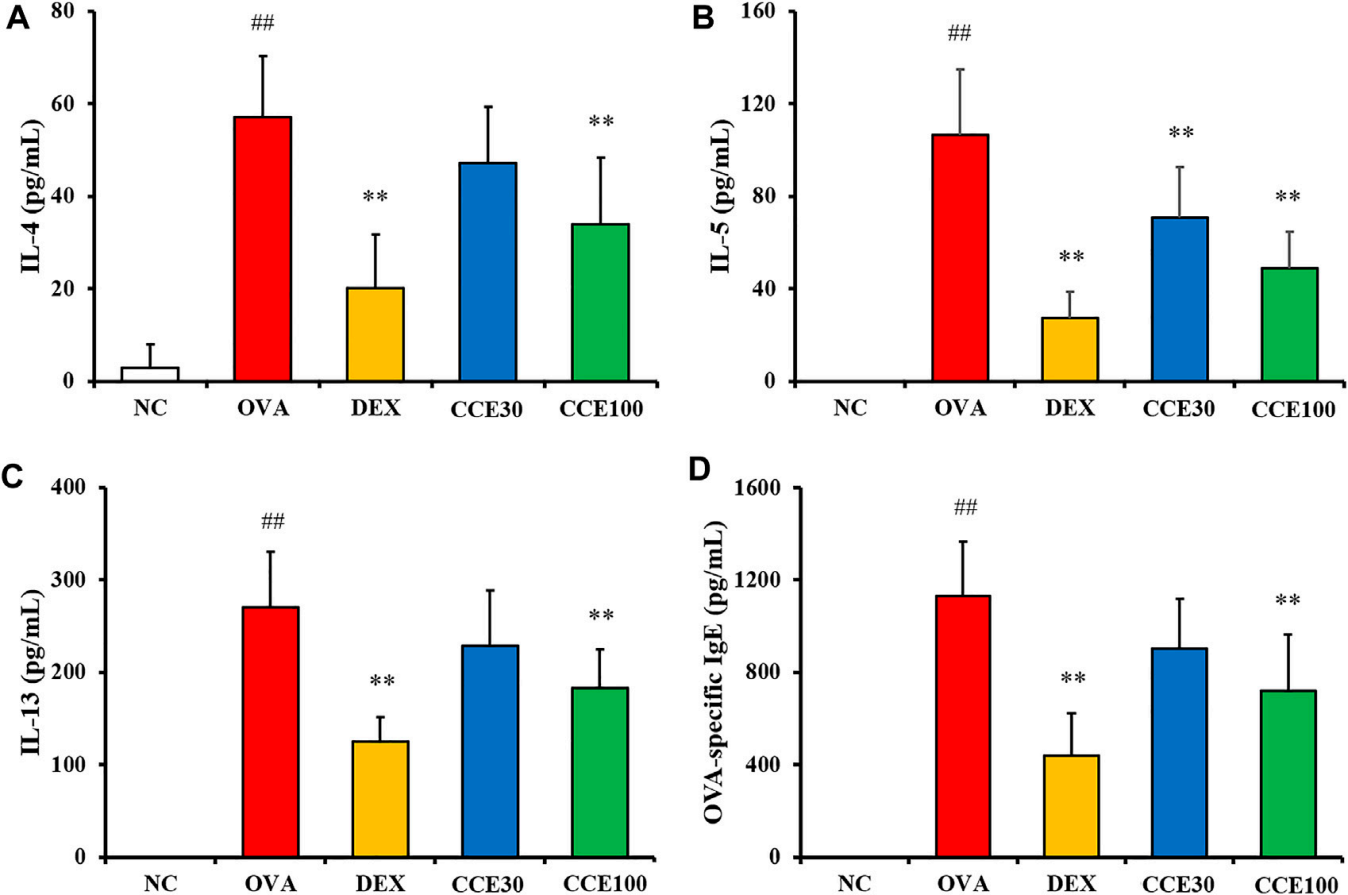
CCE declined the production of inflammatory cytokines in BALF and OVA-specific IgE in serum in OVA-exposed mice. **(A)** IL-4, **(B)** IL-5, **(C)** IL-13, **(D)** OVA-specific IgE. NC, nonasthma and PBS treatment; OVA, asthma and PBS treatment; DEX, asthma and dexamethasone treatment; CCE 30 and CCE 100, asthma and CCE treatment (30 and 100 mg/kg, respectively). ##*p* < 0.01, *vs*. NC; **p < 0.01 *vs.* OVA (*n* = 7 per group).

The OVA group exhibited a significant elevation of IL-4, IL-5, and IL-13 in comparison to the NC (Figures 4A–C). Contrastively, an obvious diminution in cytokines compared with the OVA group was observed in the DEX group. 100 mg/kg group of CCE significantly decreased the production of inflammatory cytokines in comparison to the OVA group. Although 30 mg/kg group decreased the levels of IL-4, IL-5 and IL-13, there was observed the significant difference in only IL-5 level. OVA-specific IgE levels were substantially increased in the OVA group compared with the NC (Figure 4D). But, the OVA-specific serum IgE levels in CCE groups declined, compared with the OVA group, as seen particularly in the 100 mg/kg group.

### Effects of CCE on Pulmonary Inflammation and Mucus Secretion of Lung Tissue in OVA-EXPOSED Mice

**FIGURE 5:**
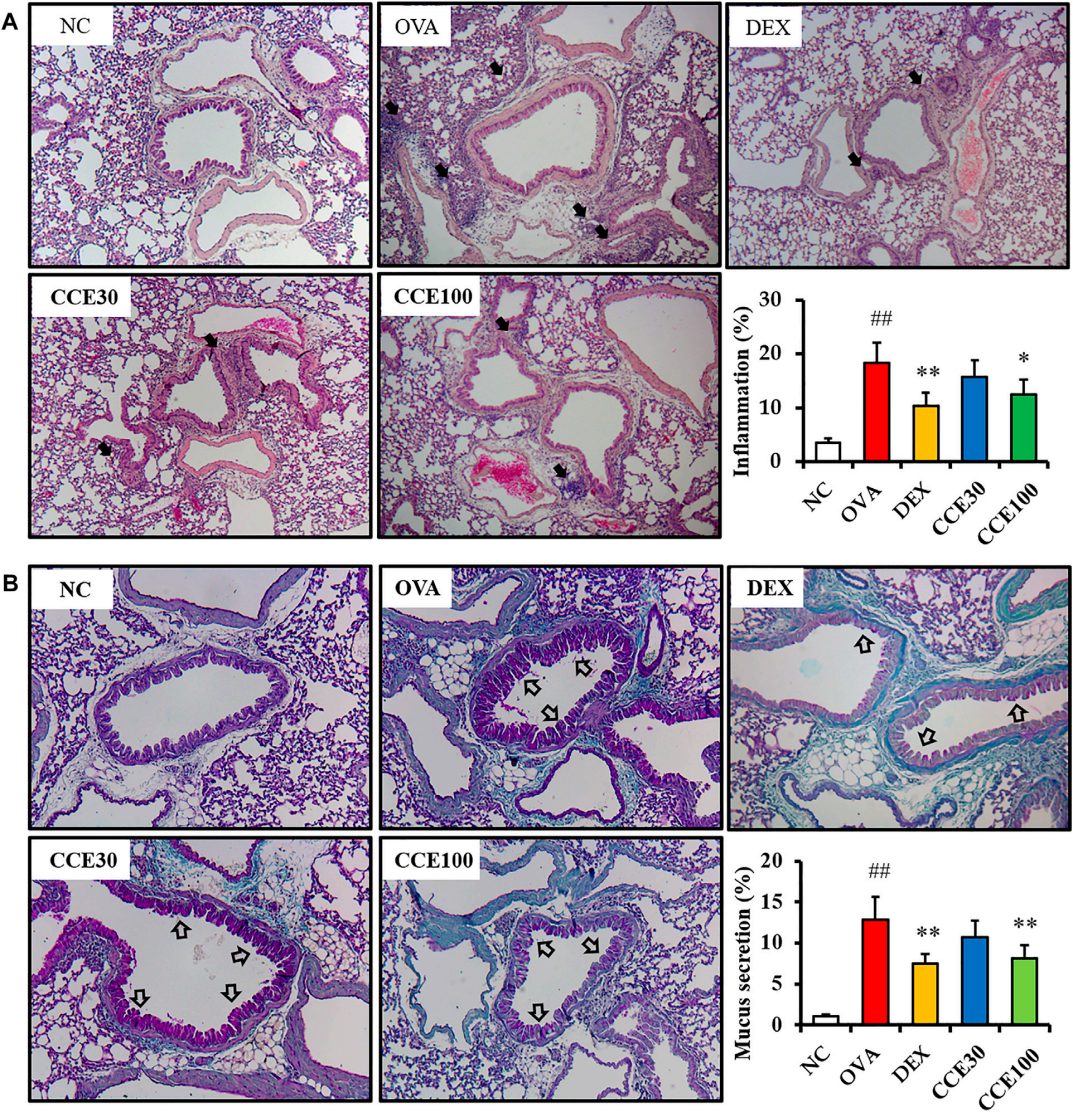
CCE inhibited inflammatory responses and mucus production in OVA-exposed mice. **(A)** Representative figure and quantification for airway inflammation, **(B)** representative figure and quantification for mucus secretion. Black arrows indicated inflammatory cell accumulation and blank arrows indicated mucus secretion. NC, nonasthma and PBS treatment; OVA, asthma and PBS treatment; DEX, asthma and dexamethasone treatment; CCE 30 and CCE 100, asthma and CCE treatment (30 mg/kg and 100 mg/kg, respectively). ##*p* < 0.01, *vs*. NC; *,**p < 0.05 and <0.01 *vs*. OVA, respectively (*n* = 7 per group).

The OVA group exhibited severe inflammatory responses in respiratory tract in comparison to the NC (Figure 5A). Contrastively, a significant diminution in inflammatory responses of respiratory tract was seen in the DEX group. Additionally, 100 mg/kg group of CCE led to a decline in the inflammatory responses of respiratory tract in comparison to the OVA group. Similarly, a substantial increase in the mucus production in the airway was detected in the OVA group, in comparison to the NC (Figure 5B). Particularly, 100 mg/kg group of CCE shown a conspicuous diminution in the mucus production in respiratory tract compared with the OVA group.

### Effects of CCE on the Phosphorylation of MAPKs in OVA-EXPOSED Mice

**FIGURE 6:**
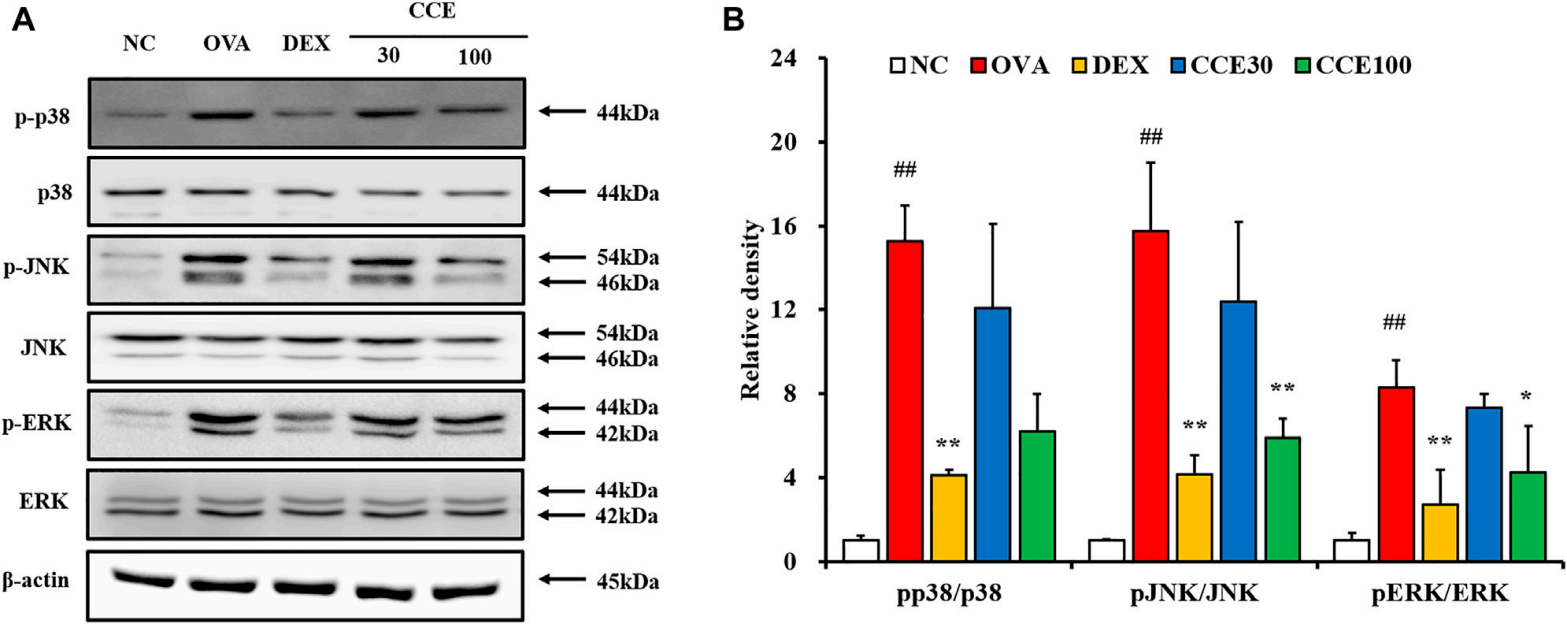
CCE reduced the phosphorylation of MAPKs in OVA-exposed mice. **(A)** protein expression on the gels, **(B)** relative density. NC, nonasthma and PBS treatment; OVA, asthma and PBS treatment; DEX, asthma and dexamethasone treatment; CCE 30 and CCE 100, asthma and CCE treatment (30 mg/kg and 100 mg/kg, respectively). ##*p* < 0.01, *vs*. NC; *,**p < 0.05 and <0.01 *vs*. OVA, respectively (*n* = 3 per group).

A steep elevation of the phosphorylation of MAPKs was seen in the OVA group, as compared with the NC (Figures 6A,B, Supplementary Figure S1). But, the DEX group displayed an obvious diminution in the phosphorylation of MAPKs compared with the OVA group. Additionally, as seen in CCE groups, CCE treatment more decreased the phosphorylation of MAPKs than the OVA group.

### Effects of CCE on MMP-9 Expression and Activity on Lung Tissue in OVA-Exposed Mice

**FIGURE 7:**
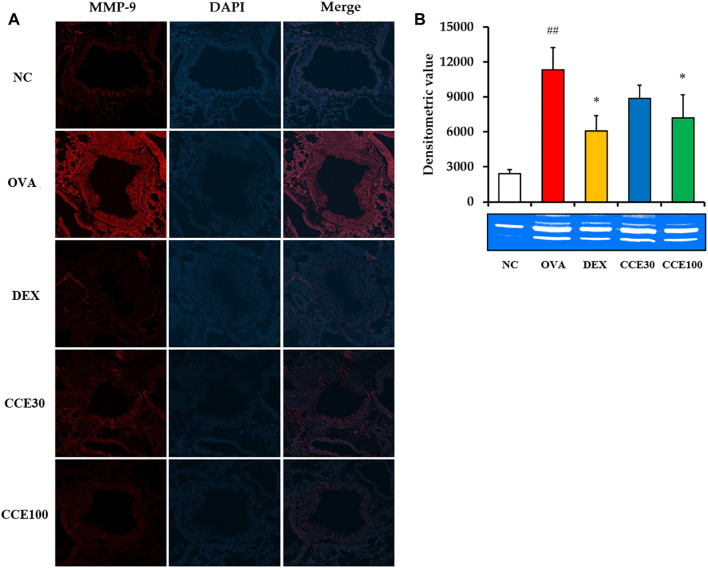
CCE decreased the expression and activity of MMP-9 in OVA-exposed mice. **(A)** Representative figure of MMP-9 expression, **(B)** gelatin zymograph. NC, nonasthma and PBS treatment; OVA, asthma and PBS treatment; DEX, asthma and dexamethasone treatment; CCE 30 and CCE 100, asthma, and CCE treatment (30 mg/kg and 100 mg/kg, respectively). ##*p* < 0.01, *vs*. NC; *p < 0.05, *vs*. OVA (*n* = 3 per group).

The gelatin zymogram showed that the MMP-9 expression and activity in respiratory tract elevated in the OVA group, compared to the NC, but obviously declined in the DEX and CCE groups, particularly in the 100 mg/kg group (Figures 7A,B).

## Discussion

The occurrence of asthma has been consistently growing worldwide with the elevation of allergens such as pollens, foods, and chemicals (Stern et al., 2020). The asthma medications that are currently in the market have limited use due to their adverse effects or low efficacy. To counteract this threat, there is a pressing need to develop remedies that treat asthma with minimal/no side effects and have high efficacy. In present study, we tested the potential of CCE to treat asthma effectively using an OVA-exposed asthma mouse model. To better understand the mechanism of action of CCE, we studied the protein expression altered by CCE treatment with a focus on MAPK and MMP-9 signaling. CCE treatment inhibits inflammatory responses of OVA-exposed animals, evidenced by the decline in inflammatory cell count, cytokines, OVA-specific IgE, and inflammatory cell infiltration in respiratory tract as seen in the histological analysis. In addition, CCE treatment led to a decrease in the phosphorylation of MAPKs and the activation of MMP-9 in respiratory tract of OVA-exposed mice.

Eosinophilia is a crucial maker of asthma (Asano et al., 2020). During the beginning of allergic asthma, contact with allergens causes T-helper type 2 (Th2) cell activation in the immune system, which produces inflammatory cytokines such as IL-4, -5, and -13 (Jung et al., 2020). These events lead to the infiltration and activation of eosinophils in respiratory tract (Brüll et al., 2016), which eventually exacerbates the airway inflammatory responses. The primary reason for this pronounced inflammatory response is the secretion of inflammatory cytokines, chemokines, and ROS due to the degranulation of eosinophils (Shin et al., 2013). Additionally, these responses stimulate bronchial smooth muscle and goblet cells, leading to increased AHR and mucus production. Histologically, these findings are characterized by an accumulation of inflammatory cell and mucus secretion in the respiratory tract (Shin et al., 2020). In this study, CCE treatment attenuated eosinophilic inflammatory responses in OVA-exposed mice, which was accompanied with a decrease in AHR, cytokines and OVA-specific IgE, supported by the histological results of reduction in recruitment of inflammatory cells and mucus secretion.

MAPKs signaling is a crucial signaling pathway involved in the pathogenesis of asthma (Johnson and Lapadat, 2002). During the development of asthma, phosphorylation of MAPKs is caused by varied stimuli, which triggers the activation of several transcription factors, resulting in the production of asthmatic mediators including cytokines, mucin, and MMPs (Alam and Gorska, 2011). This fact is backed by previous clinical and experimental studies (Liu et al., 2008; Alam and Gorska, 2011). The inhibition or deficiency of MAPKs reduces asthmatic responses due to the decrease in transcription factors (Alam and Gorska, 2011). In addition, phosphorylation of MAPKs is closely involved with the MMP-9 expression in asthmatic conditions (Shin et al., 2013). MMP-9 is a proteolytic enzyme that is associated with various pathological processes. Among its many effects, in asthmatic conditions, it destroys normal alveolar structure by degradation of substrates including collagen and gelatin that are supporting cell structures (Bajbouj et al., 2021). These reactions finally induce airway remodeling and also aggravate airway inflammation by the production of cytokines and growth factors (Shin et al., 2013). In this study, CCE treatment inhibited the phosphorylation of MAPKs in OVA-exposed mice, which was accompanied with a diminution of MMP-9 expression. These findings indicate that suppression of MAPKs and MMP-9 may form the basis of the anti-asthmatic properties of CCE.

In this experiments, effective dose of CCE on asthma was 100 mg/kg. Although 30 mg/kg of CCE exhibited the declines of asthma factors such as inflammatory cell count and IL-5, other factors did not observe the significant difference in IL-4, IL-13, OVA-specific IgE, histological analysis and protein expression. These were associated with the dose of CCE. Maybe, we considered that significant difference would have been observed if the CCE was administered at a dose of 50 mg/kg. The therapeutic effects of CCE on asthma was involved in immune modulation induced by MAPKs suppression. To prove this theory, it is considered that further experiments using genetically modified mice or inhibitors are necessary.

These anti-asthmatic effects are attributed to its bioactive ingredients including cinnamaldehyde, cinnamic acid, cinnamyl alcohol, coumarin, and 2-methoxycinnamaldehyde. Essential oils are the leading ingredients of *Cinnamomum* species and are responsible for a wide range of biological activities (Giordani et al., 2006; Mathew and Abraham, 2006; An et al., 2009; Wu et al., 2014). Five components detected in HPLC analysis make up a significant portion of the cinnamon essential oils (Wu et al., 2014). Coumarin exhibited anti-inflammatory properties in a microglia mediated inflammatory injury model *via* inhibition of MAPK signaling (Zeng et al., 2015). Cinnamic acid, cinnamaldehyde and 2-methoxycinnamaldehyde exhibited anti-inflammatory properties in various inflammatory disorders (Reddy et al., 2004; Zeng et al., 2015; Ka et al., 2016; Abozaid et al., 2020). These previous studies strongly support our belief that CCE has anti-asthmatic properties.

## Conclusion

In conclusions, CCE treatment effectively attenuated asthmatic responses including eosinophilia, AHR, cytokine production, OVA-specific IgE, eosinophilic inflammation, and mucus secretion in OVA-exposed model. These actions were closely linked with the suppression of MAPKs and MMP-9. Our study results suggest that CCE may have a potential to treat asthma.

## Data Availability

The raw data supporting the conclusion of this article will be made available by the authors, without undue reservation.

## Abbreviation

AHR, airway hyperresponsiveness; BALF, bronchoalveolar lavage fluid; CCE, *Cinnamomum cassia* (L.) J.Presl extract; ELISA, enzyme-linked immunosorbent assay; ERK1/2, extracellular signal regulated kinase 1/2; HPLC, high-performance liquid chromatography; IgE, immunoglobulin E; IL, interleukin; JNK, c-Jun N-terminal kinase; LOD, limit of detection; LOQ, limit of quantification; MAPK, mitogen-activated protein kinase; MMP, matrix metalloproteinase; OVA, ovalbumin.
