# Supplementary material for: Cinnamomum cassia (L.) J.Presl Alleviates Allergic Responses in Asthmatic Mice via Suppression of MAPKs and MMP-9
Source: Front Pharmacol. 2022 Aug 11;13:906916. doi: 10.3389/fphar.2022.906916 (PMC9405665; doi:10.3389/fphar.2022.906916)
Supplement: Supplementary file 1 [file Image1.pdf]

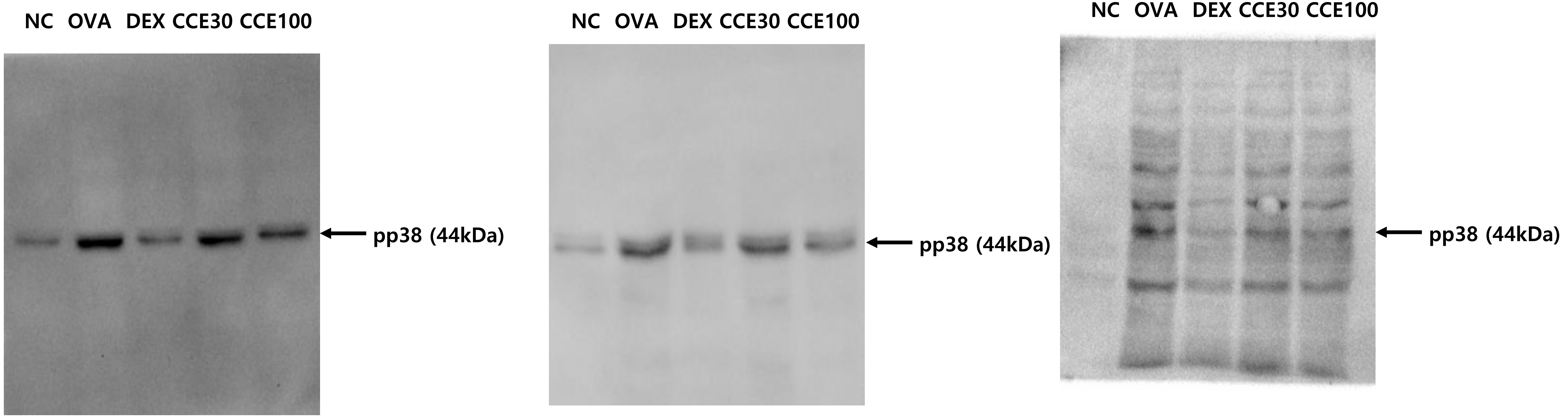

NC : Nonasthma + PBS treatment (oral gavage)

OVA : Asthma + PBS treatment (oral gavage)

DEX : Asthma + dexamethasone treatment (oral gavage)

CCE30 : Asthma + CCE treatment (oral gavage)

CCE100 : Asthma + CCE treatment (oral gavage)

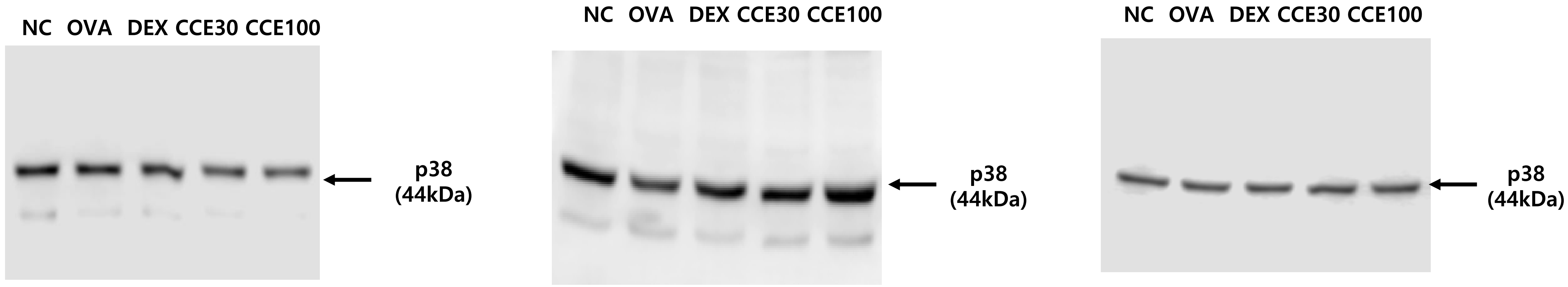

NC : Nonasthma + PBS treatment (oral gavage)

OVA : Asthma + PBS treatment (oral gavage)

DEX : Asthma + dexamethasone treatment (oral gavage)

CCE30 : Asthma + CCE treatment (oral gavage)

CCE100 : Asthma + CCE treatment (oral gavage)

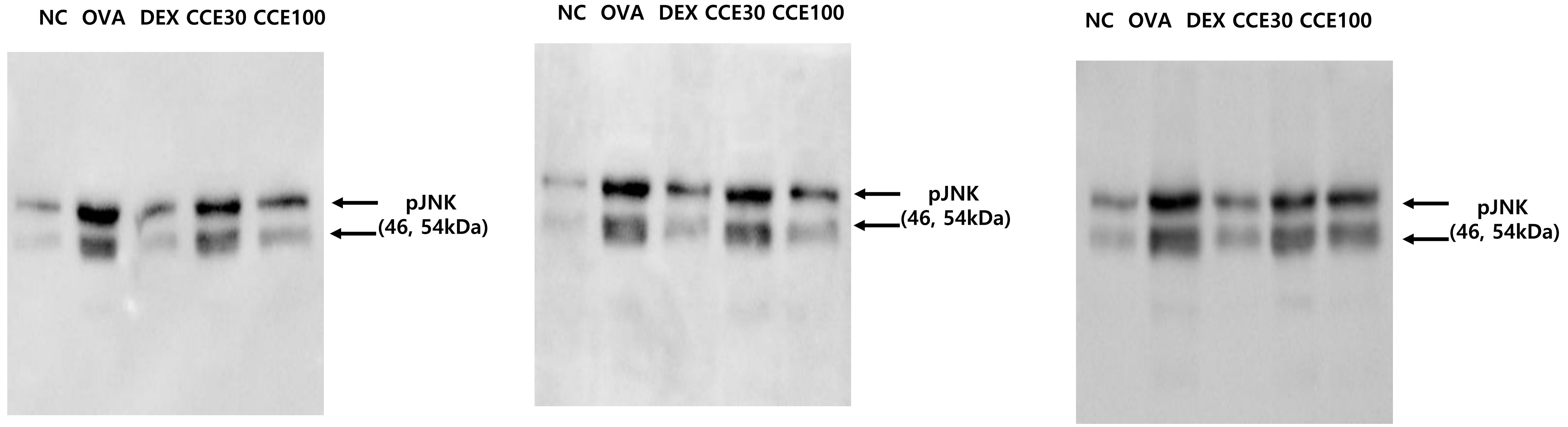

NC : Nonasthma + PBS treatment (oral gavage)

OVA : Asthma + PBS treatment (oral gavage)

DEX : Asthma + dexamethasone treatment (oral gavage)

CCE30 : Asthma + CCE treatment (oral gavage)

CCE100 : Asthma + CCE treatment (oral gavage)

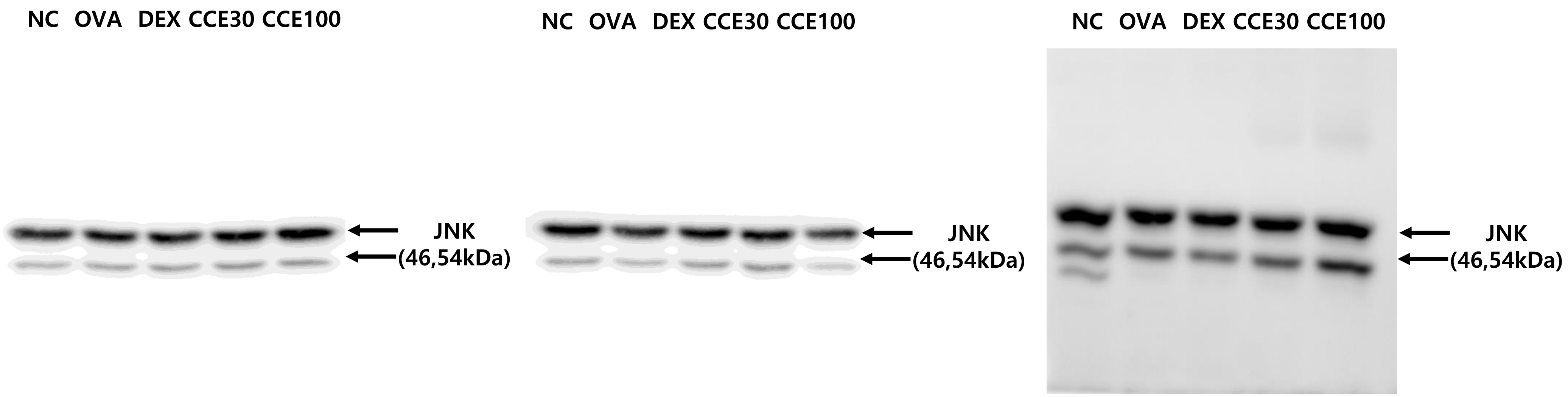

NC : Nonasthma + PBS treatment (oral gavage)

OVA : Asthma + PBS treatment (oral gavage)

DEX : Asthma + dexamethasone treatment (oral gavage)

CCE30 : Asthma + CCE treatment (oral gavage)

CCE100 : Asthma + CCE treatment (oral gavage)

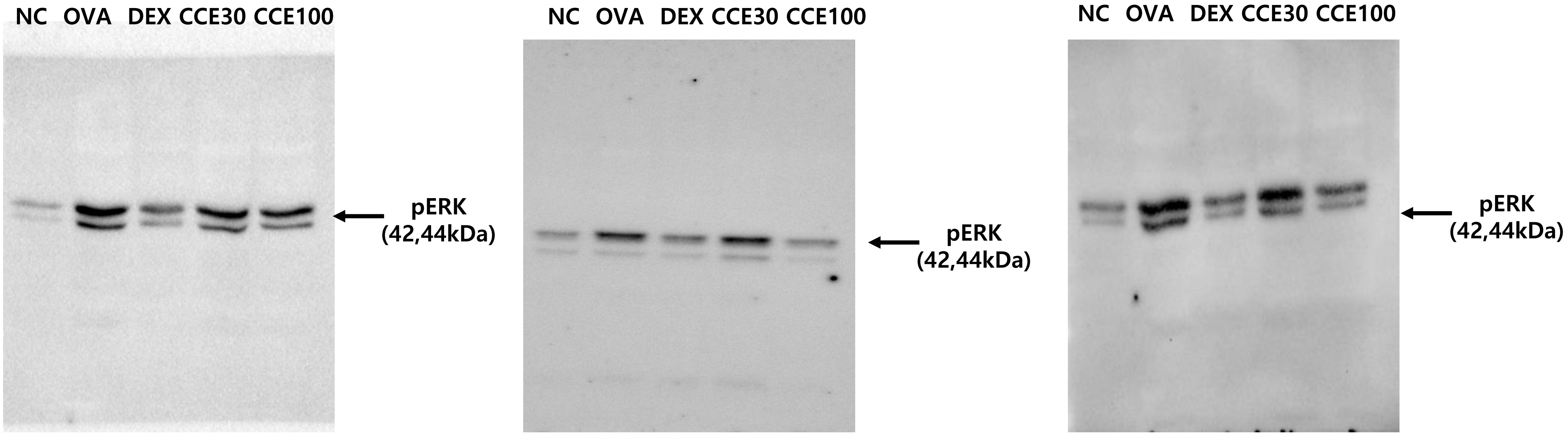

NC : Nonasthma + PBS treatment (oral gavage)

OVA : Asthma + PBS treatment (oral gavage)

DEX : Asthma + dexamethasone treatment (oral gavage)

CCE30 : Asthma + CCE treatment (oral gavage)

CCE100 : Asthma + CCE treatment (oral gavage)

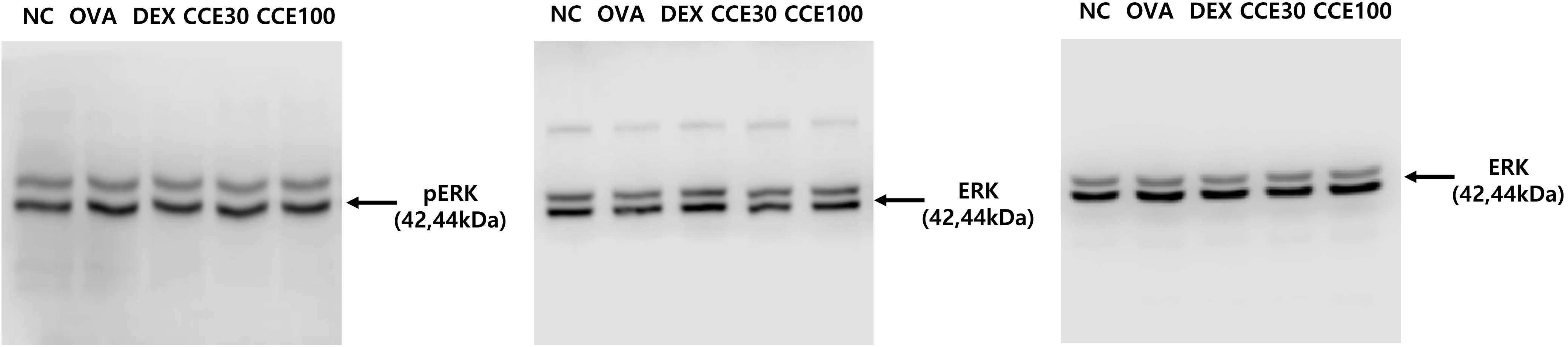

NC : Nonasthma + PBS treatment (oral gavage)

OVA : Asthma + PBS treatment (oral gavage)

DEX : Asthma + dexamethasone treatment (oral gavage)

CCE30 : Asthma + CCE treatment (oral gavage)

CCE100 : Asthma + CCE treatment (oral gavage)

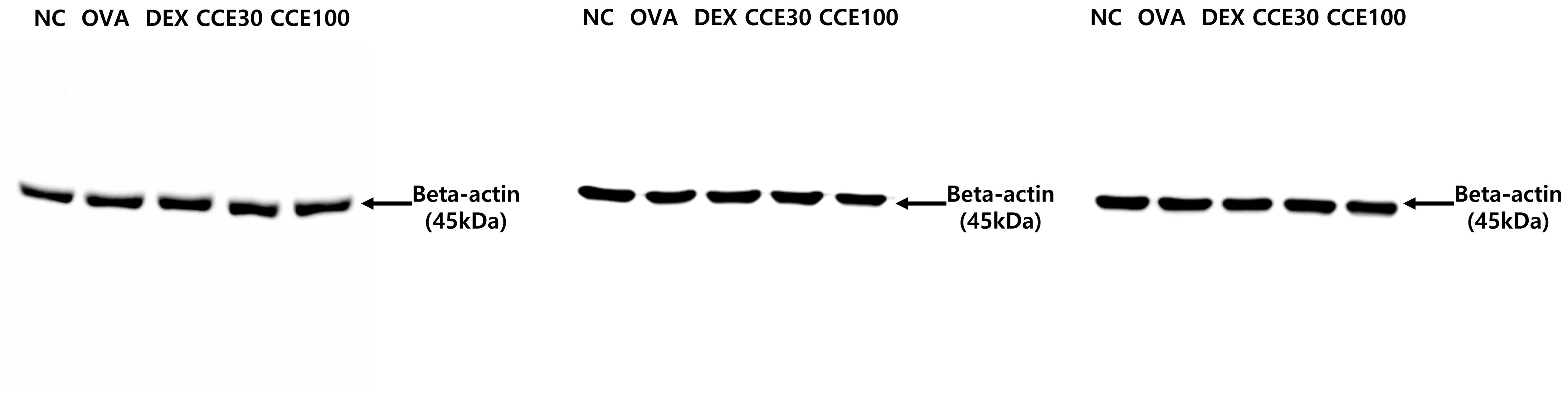

NC : Nonasthma + PBS treatment (oral gavage)

OVA : Asthma + PBS treatment (oral gavage)

DEX : Asthma + dexamethasone treatment (oral gavage)

CCE30 : Asthma + CCE treatment (oral gavage)

CCE100 : Asthma + CCE treatment (oral gavage)
